# DNA barcoding of flowering plants in Sumatra, Indonesia

**DOI:** 10.1002/ece3.4875

**Published:** 2019-01-30

**Authors:** Fitri Y. Amandita, Katja Rembold, Barbara Vornam, Sri Rahayu, Iskandar Z. Siregar, Holger Kreft, Reiner Finkeldey

**Affiliations:** ^1^ Department of Forest Genetics and Forest Tree Breeding Georg‐August University Göttingen Germany; ^2^ Research and Development Center for Environmental Quality and Laboratory; ^3^ Biodiversity, Macroecology, and Biogeography Research Group Georg‐August University Göttingen Germany; ^4^ Botanical Garden of the University of Bern Bern Switzerland; ^5^ Bogor Botanical Garden Lembaga Ilmu Pengetahuan Indonesia Bogor Indonesia; ^6^ Department of Silviculture Bogor Agricultural University (IPB) Bogor Indonesia; ^7^ University of Kassel Kassel Germany

**Keywords:** DNA marker, EFForTS project, *matK*, molecular identification, *rbcL*, Sumatra

## Abstract

The rapid conversion of Southeast Asian lowland rainforests into monocultures calls for the development of rapid methods for species identification to support ecological research and sustainable land‐use management. Here, we investigated the utilization of DNA barcodes for identifying flowering plants from Sumatra, Indonesia. A total of 1,207 *matK *barcodes (441 species) and 2,376 *rbcL* barcodes (750 species) were successfully generated. The barcode effectiveness is assessed using four approaches: (a) comparison between morphological and molecular identification results, (b) best‐close match analysis with TaxonDNA, (c) barcoding gap analysis, and (d) formation of monophyletic groups. Results show that *rbcL* has a much higher level of sequence recoverability than *matK* (95% and 66%). The comparison between morphological and molecular identifications revealed that *matK* and *rbcL* worked best assigning a plant specimen to the genus level. Estimates of identification success using best‐close match analysis showed that >70% of the investigated species were correctly identified when using single barcode. The use of two‐loci barcodes was able to increase the identification success up to 80%. The barcoding gap analysis revealed that neither *matK* nor *rbcL* succeeded to create a clear gap between the intraspecific and interspecific divergences. However, these two barcodes were able to discriminate at least 70% of the species from each other. Fifteen genera and twenty‐one species were found to be nonmonophyletic with both markers. The two‐loci barcodes were sufficient to reconstruct evolutionary relationships among the plant taxa in the study area that are congruent with the broadly accepted APG III phylogeny.

## 1 INTRODUCTION

DNA barcoding is a species identification method, using a short, standardized DNA region, so‐called DNA barcode (Hebert, Cywinska, Ball, & de Waard, 2003a). In principle, DNA barcodes contain variation that can be posed as a character to differentiate species. Although the utility of DNA barcoding for species identification has raised debates over its feasibility (Collins & Cruickshank, 2013; Krisnamurthy & Francis, 2012), the method has been increasingly applied during the last decade, especially to facilitate biodiversity studies of very diverse but taxonomically poorly known regions (Blaxter, 2004; Hajibabaei et al., 2005), such as Sumatran tropical rainforests.

Sumatran tropical rainforests are very rich in flora and fauna (Davis, Heywood, & Hamilton, 1995; Laumonier, 1997; Whitten, Damanik, Anwar, & Hisyam, 2000); nonetheless, they are only sparsely studied compared to other islands in the Malayan Archipelago (Laumonier, 1997). In terms of plant diversity, the Sumatran forests are comparable to the forests of Borneo and are richer than those found in Java and Sulawesi (Meijer, 1981). Sumatra is reported as one of the global centers of vascular plant diversity with a species density of 3,000 to 5,000 species per 10,000 km^2^ (Barthlott, Mutke, Rafiqpoor, Kier, & Kreft, 2005). Roos, Keßler, Gradstein, and Baas (2004) estimated a total number of 10,600 plant species in Sumatra with more than 300 endemic species. Laumonier (1997) argued that many scientists mistakenly consider that the flora of Sumatra is sufficiently well known since it is similar to that of the Malaysian peninsula, but many parts, especially the center of the island, are floristically unexplored territories.

Despite the importance of conserving the ecosystem, the total forest area in Sumatra has decreased from over 23 million hectares to probably less than 16 million hectares between 1985 and 1997 (World Bank, 2001). The southern provinces of Sumatra have lost most of their lowland forests, including those in protected areas (Lambert & Collar, 2002). Approximately 7.5 million hectares of primary forest loss were recorded in Sumatra during 1990–2010 and an additional 2.3 million hectares of primary forest were degraded (Margono et al., 2012). Between 2000 and 2010, the deforestation rate was estimated to be above 5% per year in the eastern lowlands of Sumatra (Miettinen, Shi, & Liew, 2011). The total deforested areas in Sumatra within 2011 alone were recorded to be approximately 2,200 hectares or as much as 3,520 soccer fields (BP‐REDD+, 2015). The causes of these massive deforestation and forest degradation are a large‐scale conversion into timber or estate crop plantations, illegal logging, and forest fires. By 2010, 3.9 million hectares of Sumatran lowland forests had been converted into oil palm (*Elaeis guineensis*) plantations (Koh, Miettinen, Liew, & Ghazoula, 2011).

The extensive loss of natural habitat puts a great number of species at risk and may lead to the loss of tropical fauna including forest‐dwelling birds (Koh et al., 2011), mammals (Maddox, Priatna, Gemita, & Salampessy, 2007), and orangutan (Gaveau et al., 2009). Undoubtedly, the destruction also affects the plant diversity (Brook, Sodhi, & Ng, 2003; Corlett, 1992; Rembold, Mangopo, Tjitrosoedirdjo, & Kreft, 2017; Turner et al., 1994). The rate of species loss in tropical forests seems to be higher than the species exploration due to lack of resources and sound species conservation management such as limited number of taxonomists working in this region, inadequate herbarium collections, and inaccessible taxonomic literature (Kiew, 2002; Meyer & Paulay, 2005; Tautz, Arctander, Minelli, Thomas, & Vogler, 2003). Species explorations become more challenging when the species cannot be identified morphologically. Identification keys based upon morphological characteristics can be difficult to use if features are not present (e.g., in sterile or juvenile specimens) or not well developed.

The use of DNA barcoding might help to overcome the limitations of morphological characters and might help to speed up species identification. This has been made possible because DNA barcoding can identify organisms at any stage of development (e.g., Barber & Boyce, 2006; Hausmann et al., 2011; Heimeier, Lavery, & Sewell, 2010; Ko et al., 2013), or at particular gender (e.g., Elsasser, Floyd, Herbert, & Schulte‐Hostedde, 2009), or specimens isolated from small and incomplete tissue, whether it is fresh, broken, or old (e.g., Hajibabaei et al., 2006; Valentini, Pompanon, & Taberlet, 2008). DNA barcoding may also help to discover new species and to identify cryptic species (e.g., Hebert, Penton, Burns, Janzen, & Hallwachs, 2004; Pauls, Blahnik, Zhou, Wardwell, & Holzenthal, 2010; Ward, Costa, Holmes, & Steinke, 2008).

DNA barcoding is now well established for animals (Crawford et al., 2013; Hebert, Cywinska, Ball, & deWaard, 2003a; Hebert, Ratnasingham, & de Waard, 2003b; Hebert et al., 2004; Lim, 2012; Nagy, Sonet, Glaw, & Vences, 2012; Ward, Zemlak, Innes, Lasr, & Hebert, 2005) by using the mitochondrial DNA *CO1* (cytochrome c oxidase subunit 1) as a standard region. However, this region is ineffective for plant identification due to generally low nucleotide substitution rates in plant mitochondria (Chase et al., 2005; Fazekas, Kesanakurti, & Burgess, 2009).

A number of candidate gene regions were suggested as potential barcodes for plants including coding genes and noncoding genes in the nuclear and plastid genomes (e.g., Chase, Cowan, & Hollingsworth, 2007; Kress & Erickson, 2007; Kress, Wurdack, Zimmer, Weigt, & Janzen, 2005; Taberlet et al., 2007). Some studies suggested DNA barcoding based on a single chloroplast region (e.g., Lahaye et al., 2008) or a combination of different regions (e.g., Chase et al., 2007; Hollingsworth et al., 2009a; Kress & Erickson, 2007). A study by Kress and Erickson (2007) showed that the various combinations of two loci were all more powerful at differentiating between species than either locus individually. In 2009, the Plant Working Group under The Consortium for Barcode of Life (CBOL) suggested that there were no other two‐loci or multi‐loci barcode provided appreciably greater species resolution than the *matK+rbcL* combination. However, in some complex groups, such as in the genus *Berberis* (Roy et al., 2010), the combination of *matK* with *rbcL* is not sufficient to distinguish all species. The investigation of these markers will contribute to the development of useful barcode information for plant identification and to document plant species globally.

This study aims to generate DNA barcodes of flowering plant species in four land‐use systems in Jambi Province (Sumatra) using two DNA chloroplast markers (*matK* and *rbcL*) and to evaluate the effectiveness of these two markers as DNA barcodes for flowering plants. Crucial characteristics for evaluating the performance of DNA barcodes include universal applicability, ease of data retrieval, and sufficient variability of the used marker (Fazekas et al., 2008; Kress & Erickson, 2007).

## 2 METHODS

### 2.1 Study sites

This study was carried out in the EFForTS project sites (https://www.uni-goettingen.de/efforts) in Jambi Province (Sumatra, Indonesia) comprises of 32 core plots sized 50 m × 50 m. Details about the EFForTS project sites and plot design are described in Drescher et al. (2016).

### 2.2 Specimen collection and identification

Herbarium specimens were collected from three individuals of as many as possible vascular plant species within the 32 core plots. The plant survey included all trees with a diameter at breast height (DBH) ≥10 cm within the entire plot and all vascular plants within five 5 m × 5 m subplots nested within each core plot. Leaf tissue (approximately 2 cm^2^) was collected from each fresh herbarium specimen and dried in silica gel for DNA barcoding analysis. Herbarium vouchers were prepared, morphologically identified, and deposited at the herbarium of the Southeast Asian Regional Centre for Tropical Biology (SEAMEO‐BIOTROP), the Herbarium Bogoriense—Research Center for Biology, LIPI, and herbarium of the University of Jambi. The results of the morphological identification were then compared to the molecular identification results. Molecular identification was conducted for all samples that were successfully barcoded, but only samples that have been morphologically identified were included in the further analysis.

### 2.3 DNA analysis

Based on the result of morphological species identification, two specimens per species were selected for genetic analysis. DNA extractions were performed on healthy dried leaf tissues from all selected samples using the DNeasy 96 Plant Kit (Qiagen, Hilden, Germany) following the manufacturer's protocols. The concentration and quality of the extracted DNA were checked by 0.8%–1% agarose gel electrophoresis with Lambda DNA as standard (Roche), visualized by UV illumination and saved using a polaroid camera.

Each extracted DNA was amplified by performing polymerase chain reaction (PCR) using universal primers listed in Table 1. For rbcL, the amplification was straightforward, while for matK, two different amplification reactions were performed. First, the DNA of all investigated samples were amplified using the universal primer pair 1RKIM_f and 3FKIM_r (Table 1). The second amplification reaction, using the primer pair 390f and 990r (Table 1), included only those samples which showed no amplification product or produced multiple PCR products in the first amplification reaction.

**Table 1:** Universal primers of *matK* and *rbcL* used in DNA amplification and sequencing

No.	Region	Name of primer	Primer sequence (5′ → 3′)	References
1	*matK*	*3F_KIM_f*	CGTACAGTACTTTTGTGTTTACGAG	Ki‐Joong Kim (unpublished)
		*1R_KIM_r*	ACCCAGTCCATCTGGAAATCTTGGTTC	Ki‐Joong Kim (unpublished)
		*390f*	CGATCTATTCATTCAATATTTC	Cuenoud et al. (2002)
		*990r*	GGACAATGATCCAATCAAGGC	Dayananda, Ashton, Williams, & Primack (1999)
2	*rbcL*	*rbcLa_f*	ATGTCACCACAAACAGAGACTAAAGC	Krees and Erickson (2007)
		*rbcLa_r*	GAAACGGTCTCTCCAACGCAT	Fazekas et al. (2008)

The sequencing reactions were performed using the ABI PrismTM Big DyeTM Terminator Cycle Sequencing Ready Reaction Kit v1.1 (Applied Biosystems), based on the principles described by Sanger, Nicklen, and Coulson (1977). Data were collected from capillary electrophoresis on an ABI Prism 3100® Genetic Analyzer with the Sequence Analysis Software v3.1 (Applied Biosystems). The sequencing was performed with the same primers used for amplification in both directions. The amplification and sequencing reaction mixtures are shown in Supporting Information Appendix 1, while the temperature profiles of the PCR for amplification and sequencing are shown in Supporting Information Appendix 2.

### 2.4 Sequence analysis

To ensure the generated DNA barcodes were as accurate as possible, sequence editing was performed using CodonCode Aligner software (CodonCode Corporation, Dedham, USA). Furthermore, each of these edited barcodes was assigned to a particular taxon by comparing it with the nucleotide sequences in GenBank database and Barcode of Life Database (BOLD).

Moreover, the results of sequence identification were cross‐checked with the morphological identification results. The match between morphological and molecular identification results was counted into three levels: species, genus, and family. The following decisions were made for correct identification assignments, namely: (a) when the species name from the molecular identification matched the species name from the morphological identification, then it was counted as a correct species identification, (b) when the identification result only matched the genus or family, then it was counted as correct genus or family identification, and (c) when the result between morphological and molecular identification did not match, it was counted as incorrect identification if *matK* and *rbcL* both showed similar results at least at family level, or it was counted as mislabeling/contamination if the results of *matK* and *rbcL* were different. Herbarium specimens were double‐checked in cases of incorrect identification.

Sequence alignment was carried out independently for each marker in two stages. First, multiple sequences were aligned according to their families using the ClustalW program (Thompson, Higgins, & Gibson, 1994) embedded in MEGA6 (Tamura, Stecher, Peterson, Filipski, & Kumar, 2013). Reference sequences were downloaded from GenBank/BOLD and included in the alignment for those species represented with only one sample. The alignment results were subsequently checked for the occurrence of ambiguities caused by the presence of indels and/or substitutions and edited if necessary. In the second stage, all aligned sequences from each family were manually aligned with sequences from other families. Gaps were added if necessary, and the final alignment was trimmed at both ends. The aligned sequences of *rbcL* and *matK *were combined to obtain two‐loci DNA barcodes using SequenceMatrix software (Vaidya, Lohman, & Meier, 2011).

Identification success was also calculated with best‐close match analysis as implemented in TaxonDNA (Meier, Kwong, Vaidya, & Ng, 2006). This analysis only included the species with at least two representatives. A threshold value T was determined for each dataset as a divergence percentage in which 95% of all intraspecific distances were found. In this method, all recovered barcodes were formatted as both database and query. A query can only be identified if the corresponding sequence has a match in the dataset that falls into the 0% to T% interval. If the species name was identical, the query was considered to be successfully identified. A query was considered ambiguously identified when it matched more than one sequence of different species besides the correct species. On the other hand, a query was considered incorrectly identified when it matched to sequences belonging to other species. All queries without such a match would remain unidentified.

Pairwise distance matrices were created to calculate the genetic distance using MEGA6 (Tamura et al., 2013) based on the Tamura‐Nei model (1993) assuming the differences in substitution rate between nucleotides and the inequality of nucleotide frequencies with gamma‐distributed rates between sites and the pattern between lineages were assumed to be heterogeneous. The calculation results of intra‐ and interspecific divergences in these matrices were separated using ExcaliBAR (Aliabadian et al., 2014) to facilitate the measures of distance range and distance mean of each type of divergence. Frequency (%) distribution of intra‐ and interspecific divergences of each marker was calculated and depicted in graphics using Excel to find possible “gap” between these two divergences. This so‐called barcoding gap illustrates the effectiveness of DNA barcodes in discriminating query species from one to another. An ideal barcode can be determined by the presence of a barcoding gap, which occurs when the minimum value of the interspecific divergence is higher than the maximum level of intraspecific divergence (Meyer & Paulay, 2005).

Based on the aligned sequences, phylogenetic trees were reconstructed using MEGA6 (Tamura et al., 2013) with three different algorithms: maximum parsimony (MP), maximum likelihood (ML), and neighbor joining (NJ). Percentages of species, genus, and family monophyletic clades were calculated from each reconstructed tree. Furthermore, ordinal‐level phylogenies were reconstructed based on maximum likelihood trees of each used marker and were compared to APG III (APG III 2009) phylogenies to see if there were inconsistencies between these two topologies.

## 3 RESULTS

From all 5,328 samples collected from the field, only 2,590 samples were included in the study due to time restriction. The selection of studied samples was based on the consideration to involve as much species as possible, and each of these species should be represented at least by two samples. Species with only one sample were still included, but the barcodes generated from single‐sampled species were excluded from the pairwise analysis.

We extracted DNA from dried leaf specimens without noticeable difficulties. The amplification and sequencing, however, turned out to be more problematic especially when using *matK *primers. Recoverability of DNA sequences for *rbcL* was overall high (amplification and sequencing success were 96.9% and 94.7%, respectively). The amplification and sequencing results using the primer of *matK* were only moderately successful (79.1% and 65.8%, respectively). A total of 1,207 *matK* barcodes representing 441 species of 97 families of 40 orders, and 2,376 *rbcL* barcodes representing 750 species of 126 families of 44 orders, were generated in this study.

For both markers, the highest match between morphological and molecular identification was at genus level (46.6% with *matK* and 51.3% with *rbcL*). The matched identification at species level was higher with *matK* than with *rbcL* (30.2% and 22.4%, respectively). Meanwhile, incorrect identification was relatively low for both regions (3.5%). To maintain the accuracy of the analysis, we excluded all misidentified or presumably mislabeled barcodes from the dataset. Since the study aims at comparing the performance of *matK* and *rbcL* and to generate two‐loci barcodes, only samples from which both *matK* and *rbcL* barcodes were successfully recovered were included in the further analysis. Consequently, only 322 samples from 161 species (two samples per species) were included in best‐close match and barcode‐gap analysis and 334 samples from 334 species (one sample per species) were included in phylogenetic analysis.

According to the best‐close match analysis, *matK* has higher overall species identification success compared to *rbcL* (78.3% and 71.4%, respectively), and the highest correct species identification was obtained by the combination of both markers (81.1%). There were 22 species which remained unidentified by each marker and the two‐loci marker.

**Figure 1:**
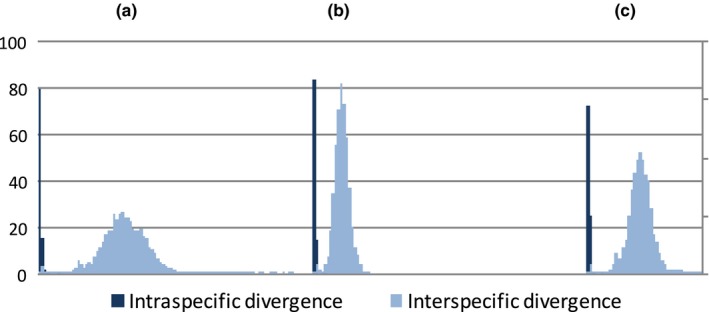
Frequency (%) distribution of intraspecific and interspecific divergences of pairwise sequences of matK (a), rbcL (b), and matK+rbcL(c)

Furthermore, this study showed that the mean value of intraspecific divergences (0.0008–0.0014) was very low and the mean value of the interspecific divergences (0.1–0.3) was significantly higher (unpaired *t*‐test, *p* < 0.01). The frequency (%) distribution of intraspecific and interspecific divergence using three markers (Figure 1) showed that no barcode gaps existed as the intraspecific divergences overlapped with interspecific divergences.

As expected, *matK* had a higher discrimination level than *rbcL* (80% and 73%, respectively) but the difference was not significant (one‐way ANOVA, *p* > 0.05). The combination of *matK* and *rbcL* improved the discrimination up to 89%. Forty‐four out of 161 species could not be discriminated by *rbcL* and eleven of them were not discriminated by any of the markers including the two‐loci barcode. These species were mostly from species‐rich genera, such as *Ficus *(Moraceae), *Santiria *(Burseraceae), and *Litsea *(Lauraceae).

Nine phylogenetic trees (Supporting information Appendix 3–11) were constructed based on multiple sequence alignments of *matK*, *rbcL*, and *matK+rbcL* using three different methods: maximum parsimony (MP), neighbor joining (NJ), and maximum likelihood (MP). Each tree was observed and similar topologies were found amongst these trees (Table 2).

**Table 2:** Percentage of monophyletic clades recovered in nine reconstructed phylogenetic trees

Barcode	Monophyletic with support value >70%								
	Maximum Parsimony (MP)			Neighbor Joining (NJ)			Maximum Likelihood (ML)		
	Family	Genus	Species	Family	Genus	Species	Family	Genus	Species
***matK***	95.9	68.4	73.9	93.9	66.7	69.6	98.0	64.9	68.9
***rbcL***	95.9	63.2	60.3	93.9	63.2	64.0	89.9	63.2	55.9
***matK+rbcL***	100.0	71.9	73.3	100.0	64.9	73.9	100.0	70.2	75.2

Seventeen families were not included in the calculation of family‐level monophyletic percentage as these families were presented with only one taxon. The two‐loci marker provided 100% taxonomic resolution at family level with all three different methods. Twenty‐two species were nonmonophyletic in all phylogenetic trees (Supporting information Appendix 12). The nonmonophyletic species mostly originated from species‐rich families, such as Burseraceae, Myristicaceae, Moraceae, Phyllanthaceae, Lauraceae, Sapindaceae, and Annonaceae.

**Figure 2:**
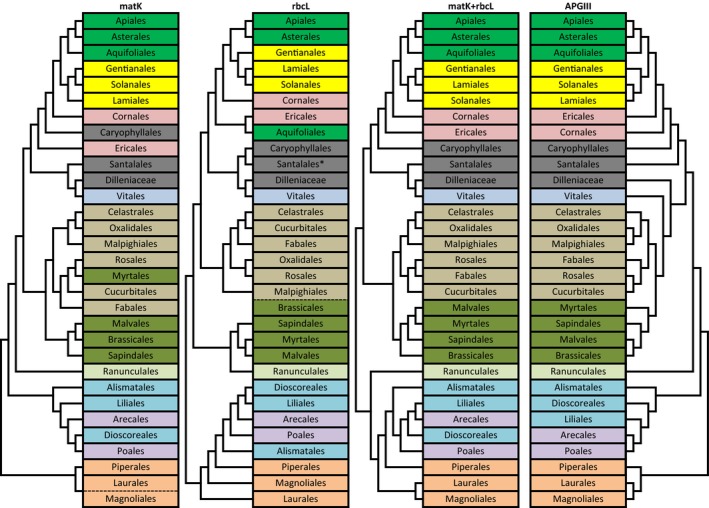
Comparison between ordinal‐level phylogeny of flowering plants based on DNA barcodes and APG III (2009). The dash lines indicate that the two orders are not clearly separated. ^*^Santalales in rbcL phylogeny tree is a nonmonophyletic clade

The ordinal‐level phylogeny of flowering plants shows the relationship between orders of flowering plants and the grouping of these orders (Figure 2). The matK marker misplaced Myrtales and failed to separate Laurales from Magnoliales. Meanwhile, the rbcL marker misplaced Aquifoliales and grouped Malpighiales and Brassicales into one monophyletic clade. This marker also failed to make Santalales a monophyletic clade. However, this marker successfully separated Laurales from Magnoliales. Finally, the combination of matK and rbcL improved the topologies of the tree and put nearly all orders into the right position compared to APG III phylogeny.

## 4 DISCUSSION

### 4.1 Recoverability and quality of *matK* and *rbcL* barcodes

The *rbcL* universality as DNA barcode observed in this study confirms that DNA sequences could be easily obtained with *rbcL* primers from a wide range of tropical plant species (e.g., Gonzales et al., 2009; Lahaye et al., 2008; Parmentier et al., 2013). In contrast to *rbcL*, *matK* seems to be less suitable for tropical floras compared to temperate one (e.g., Bruni et al., 2012; de Vere et al., 2012; Gonzales et al., 2009). This might be due to higher evolutionary rates in tropical compared to temperate plants (Gillman, Keeling, Gardner, & Wright, 2010). The PCR of *matK* performed in this study was using two pairs of primers which were found to be effective to generate DNA barcodes from specific taxa, such as *Tetrastigma* (Fu, Jiang, & Fu, 2011), *Hedyotis *(Guo, Simmons, But, Shaw, & Wang, 2011), or Asteraceae (Gao et al., 2010). These primers, however, became less effective when they were used for a wide range of species (Gonzales et al., 2009; Kress et al., 2010). A certain primer pair did not always yield a PCR product in all members of a group of seemingly closely related taxa, indicating that the primers themselves are not conserved.

The use of *matK* as a barcode has been criticized mainly because universal primers are not available (e.g., Bafeel et al., 2011; Dong et al., 2015). A study by Fazekas et al. (2008) showed a relatively high rate of sequencing success for this marker after using up to 10 primer pairs. The usefulness of *matK* primers is proven when they are used in specific species or taxa, such as *Camellia sinensis* (Stoeckle et al., 2011), Lamiaceae (De Mattia et al., 2011), or palms (Jeanson, Labat, & Little, 2011). In a review of the best barcode for plants, Hollingsworth, Graham, and Little (2011) indicated that *matK* still needs optimization in regard to primer combinations and needs to be adapted to specific taxonomic groups.

### 4.2 Plant species identification success using *matK* and *rbcL*

As one way to evaluate the success rate of species identification, we compared the results from morphological identification with the results from molecular identification. Some authors suggested a superiority of molecular identification in comparison with morphological identification (Newmaster, Ragupathy, & Janovec, 2009; Stace, 2005). However, this study showed that DNA barcoding alone is not sufficient to assign all DNA sequences to a correct species name. Only 22%–30% of the samples were correctly assigned to the correct species, while the majority of correct identifications was limited to genus level (46%–51%).

Approximately three percent of mismatch between morphological identification results and DNA identification results were found in this study that could be due to several reasons. A specimen could be misidentified when it was found to have the highest similarity to a reference sequence that was falsely identified. The mismatch between morphological and molecular identification could also happen when the taxonomist misidentified the voucher. Morphological identification is difficult in the absence of certain features, such as flowers or fruits, especially when dealing with species‐rich groups. A high percentage of nonfertile material is particularly common in ecological projects such as ours. In the case of incorrect morphological identification, the herbarium vouchers of corresponding samples should be verified morphologically once again.

The success of species identification using DNA barcoding depends very much on the taxa in question, as much as the utilized marker. For example, in this study, the family Piperaceae resulted in high species‐matched identification when using *matK* (60%) but no success at all when using *rbcL*. Meanwhile, for the family Asteraceae, the species‐matched identification was higher with *rbcL* (50%) than with *matK* (30%).

Another factor affecting the success of species identification using DNA barcoding is the availability of nucleotide data of the corresponding taxa in the DNA sequences database such as GenBank and BOLD. Through this study, 303 newly barcoded tropical plant species have been uploaded to BOLD. Forty‐one percent of the 772 species investigated in this study still had no nucleotide data in BOLD and Genbank. Thus, a significant proportion of samples belonging to species which were not yet recorded in the reference databases lead to increased rates of unassigned samples. Incorrect specimen assignment is more often due to the incompleteness of molecular datasets rather than the data analysis (Bruni et al., 2010; Burgess et al., 2011; Cowan & Fay, 2012). An accurate and complete molecular database, especially for plant species, is still far from being achieved in the present state. Such a database will hopefully be developed in the future as many studies and projects of plant DNA barcoding are going on (e.g., http://botany.si.edu/projects/DNAbarcode/intro.htm; http://xmalesia.info/index.html).

### 4.3 Discriminatory power of *matK* and *rbcL*

None of the markers used in this study successfully obtained a DNA barcoding gap. All of the minimum values of interspecific divergence obtained from three different markers were lower than the maximum values of intraspecific divergence. In studies of DNA barcoding of specific plant taxa, for example, *Ludwigia *(Ghahramanzadeh et al., 2013), *Abies,* *Cupressus* (Armenise, Simeone, Piredda, & Schirone, 2012), and *Tetrastigma* (Fu, Jiang, & Fu, 2011), the distribution of intra‐ versus interspecific distances was relatively well separated. Meanwhile, large‐scale plant diversity inventories (Lahaye et al., 2008; Parmentier et al., 2013) reported the absence of barcoding gaps by using a combination of potential markers. The richness of the dataset might have contributed to the wider distribution of the intra‐ and interspecific divergences which then increase the possibility of them to overlap. This implies that the sampling intensity and variety would influence the distribution of the intra‐ and interspecific variation within the dataset.

Despite the absence of barcoding gaps, the barcodes generated in this study have relatively high discriminatory power. According to Hollingsworth et al. (2011), most of the plant barcodes would have discriminatory power of more than 70%. Studies by Kress et al. (2009) and Burgess et al. (2011) showed that barcoding of distantly related taxa typically results in high levels of discriminatory power.

The *matK*+rbcL marker has the highest number of discriminated species compared to *matK* or *rbcL* alone. This is because the use of two‐loci barcodes maximized the genetic variation, thus minimizing the number of identical barcodes between different species. All species that could not be discriminated have barcodes identical to other species from the same family. Identical barcodes across different genera of the same family were uncommon with *matK* but more common with *rbcL*. However, *matK* and *rbcL* mostly failed to discriminate different species from the same genus. These two plastid markers are therefore not variable enough to be effective barcodes for closely related species in certain taxa.

To improve the analysis of closely related taxa, noncoding plastid genes, such as *trnH‐psbA*, could be used as an additional marker (Hollingsworth et al., 2011). A study by Kress and Erickson (2007) showed that *trnH‐psbA* has dramatically higher sequence variability than the coding genes because it has a higher number of single‐nucleotide polymorphisms (SNPs). Hence, *trnH‐psbA* can be a suitable marker to discriminate among closely related species. Moreover, nuclear genomic regions, such as the internal transcribed spacer (ITS) region, were suggested as potential DNA barcodes by Kress et al. (2005). ITS sequences generally show high levels of interspecific sequence variability (Cowan & Fay, 2012) and has been used successfully to classify angiosperms (Li et al., 2011).

### 4.4 The phylogeny of flowering plants of Jambi based on *matK* and *rbcL*

Both *matK* and *rbcL* showed high family‐level resolution, and the combination of *matK* and *rbcL* succeeded to resolve all of the families into monophyletic clades with high bootstrap value. Furthermore, the taxonomic resolution at the genus level was much lower compared to the family level which was expected. Surprisingly, the genus‐level monophyletic percentages were found slightly lower compared to the species level in all trees, except for MP and ML trees using *rbcL*. A similar study by Gonzalez et al. (2009) reported larger numbers of monophyletic genera compared to monophyletic species. This difference can be explained by the fact that the proportion of distantly related species included in the dataset in this study was higher than the proportion of closely related species. Thus, the probability of resolving monophyletic‐species clades was higher than to resolve the monophyletic‐genus clade. Finally, the species‐level resolution in this study is comparable to similar studies (Gonzalez et al., 2009; de Vere et al., 2012). However, the two‐loci barcode did not improve the species‐level resolution significantly. Combining these two chloroplast markers was not sufficient to provide 100% of species monophyly.

Of 76 families included in the phylogenetic tree reconstruction, Burseraceae and Phyllanthaceae were the families with the highest number of unresolved genera. Most of the species in these genera were found to have identical sequences, so they could not be separated from each other. Identical sequences between species of different genera could be common if the marker was not variable enough, such as *matK* and *rbcL*. In this study, it was revealed that *matK* and *rbcL* were not sufficiently variable for species‐rich groups.

The phylogenetic trees based on the *rbcL* marker resulted in larger numbers of unresolved species than *matK*. At least eighteen species were nonmonophyletic according to *rbcL* but monophyletic according to *matK*. The unresolved species found in this study could be explained by two reasons. First, these species might have identical genetic information with other species belonging to the same genera/family. Second, these species might have higher intraspecific than interspecific divergence; thus, they were grouped with the allospecies but not with the conspecies.

A number of constraints are limiting DNA barcoding of plant species including slow evolution rates (Palmer et al., 2000) and high incidence of hybridization (Knobloch, 1972). The genetic variation caused by hybridization cannot be simply detected by plastid markers (Fazekas et al., 2008, 2009). Nevertheless, none of the plant DNA markers are perfect in every case (Hollingsworth et al., 2011). Indeed, one of the future challenges for plant DNA barcoding is to find the most suitable marker to tackle these problems. As the DNA sequencing technology and bioinformatic tools are progressively advancing, the development of new primers will be much easier and at the end will increase the success of DNA barcoding. The application of next‐generation sequencing (NGS) technology will enhance the capability of DNA barcoding as a powerful tool in the studies of ecology, evolution, and conservation biology (Kress, Garcia‐Robledo, Uriarte, & Erickson, 2014).

## 5 CONCLUSION

We conclude that the two plastid markers *matK* and *rbcL* as plant barcodes work reasonably well in identifying flowering plant species in Sumatran lowland rainforest and surrounding agricultural systems, at least up to genus level. However, there are taxa that are difficult to be distinguished using *matK* and *rbcL*. These taxa mostly belong to species‐rich clades with low interspecific divergences. DNA barcoding of closely related species results in low success, especially when using coding plastid markers, such as *matK* and *rbcL*.

The success of species identification strongly depends on the availability of an accurate and complete molecular database. Such database should include sufficient barcodes for each species distributed over its entire distribution range to cover the full range of its intraspecific variability. Thus, future studies ideally include all congeneric species from a geographic region and maximize the geographic diversity of samples for each species. Moreover, utilization of supplement markers, such as *psbA‐trnH* or ITS, is highly recommended in combination with *matK* and *rbcL*.

All of DNA barcodes generated in this study, comprises more than 500 species of flowering plants, are uploaded to BOLD. This, coupled with the collection of herbarium vouchers, will improve the usability of DNA barcodes for plant identification.

## AUTHOR CONTRIBUTIONS

F.Y.A. performed specimen collection, laboratory work, sequence analyses and wrote the manuscript. K.R. performed specimen collection, morphology identification and provided critical review of the manuscript. B.V. supported part of the laboratory work and data analysis, and revised the manuscript. S.R. provided author citation for each botanical name of species barcoded in this study and revised the manuscript. I.Z.S. provided the sample collection permit and mutual transfer agreement (MTA) documents, and revised the manuscript. H.K. and R.F. supervised the research and revised the manuscript.

## Data Availability

All data for the project were managed in the BOLD database in a project called “DNA Barcoding of Vascular Plants in Jambi, Indonesia” (project code CRCZ). A list of all species barcoded in this study is available as Supporting Information (Table S1).
